# Smart Biomechanical Adaptation Revealed by the Structure of Ostrich Limb Bones

**DOI:** 10.3390/biomimetics8010098

**Published:** 2023-02-28

**Authors:** Simone Conti, Giuseppe Sala, Octavio Mateus

**Affiliations:** 1 GEOBIOTEC, Department of Earth Sciences, NOVA School of Science and Technology, Campus de Caparica, 2829-516 Caparica, Portugal; 2 Department of Aerospace Science and Technology, Politecnico di Milano, Via La Masa 34, 20156 Milano, Italy

**Keywords:** ostrich, biomimesis, structural adaptation, mechanical performances, material properties

## Abstract

Ostriches are known to be the fastest bipedal animal alive; to accomplish such an achievement, their anatomy evolved to sustain the stresses imposed by running at such velocities. Ostriches represent an excellent case study due to the fact that their locomotor kinematics have been extensively studied for their running capabilities. The shape and structure of ostrich bones are also known to be optimized to sustain the stresses imposed by the body mass and accelerations to which the bones are subjected during movements. This study focuses on the limb bones, investigating the structure of the bones as well as the material properties, and how both the structure and material evolved to maximise the performance while minimising the stresses applied to the bones themselves. The femoral shaft is hollowed and it presents an imbricate structure of fused bone ridges connected to the walls of the marrow cavity, while the tibial shaft is subdivided into regions having different mechanical characteristics. These adaptations indicate the optimization of both the structure and the material to bear the stresses. The regionalization of the material highlighted by the mechanical tests represents the capability of the bone to adapt to external stimuli during the life of an individual, optimizing not only the structure of the bone but the material itself.

## 1. Introduction

This study presents the evolutionary adaptations of ostrich limb bones, investigating the material properties and highlighting that the shape, structure and even the material of which they are made is adapted to optimize the mechanical performance. It explains the evolutionary adaptations of the bone structures in response to the biomechanical conditions imposed by being the fastest bipedal animal alive on Earth. It is often cheaper to optimize the structure by changing its shape rather than modifying the material of which the object is composed; yet, here we present that not only are ostrich bone structures optimized to bear the loadings, but also the bone material itself is adapted to maximise the performance. The experimental approach was chosen to gather new data to compare with the data collected from the literature. The aim is to describe the mechanical characteristics of different regions within ostrich femora and tibiae and correlate them, taking into account the shape and structure of the whole bone and the biomechanical behaviour in response to the loadings normally working on the ostrich limb.

### 1.1. Why Ostrich?

**Figure 1:**
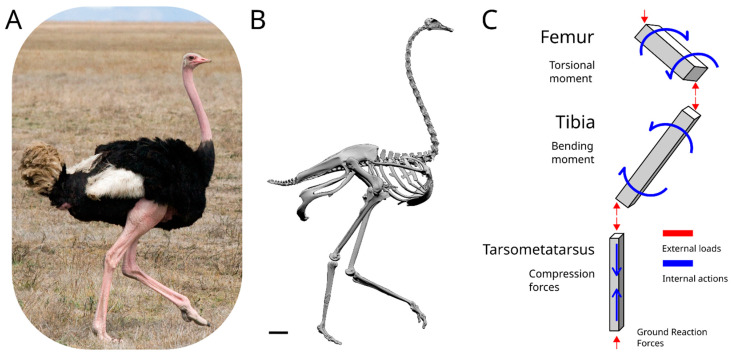
Scheme of the acting forces on a running ostrich. (**A**) Photo of an ostrich, courtesy of Mr. Stig Nygaard. (**B**) 3D model of an ostrich, courtesy of the Idaho Virtualization Laboratory. The scale bar is equal to 10 cm. (**C**) Simplified model of a standing ostrich leg. The elements are simplified in beams, with the distribution and direction of the external forces acting on each element illustrated in red, while the simplified and most important internal actions of each bone are represented in blue.

Large ratites, such as the ostrich, *Struthio camelus* Linnaeus 1758, or the emu, *Dromaius novaehollandiae* (Latham, 1790), are the largest birds alive and have become a reference point in the research on various ambits, from biodynamics to veterinary. Their vast availability is related to the food industry, making these animals ideal to study their physiology and dynamics of motion. Ostriches and emus are among the fastest-running animals with exceptional stamina, and their dynamics of motion have been the subject of many studies, aimed to comprehend their capabilities [1,2,3,4,5,6,7,8,9,10]. Birds are obligate bipedal creatures, like humans, and ostriches have comparable masses to humans, making them an ideal comparison for our species [3,11]. Studies on ostrich and emu dynamics have been conducted in parallel to the study of bone material properties, aimed mainly at establishing the generic values of mechanical properties for bone [2,12,13,14]. Locomotion has been subjected to extensive studies, correlating the function of musculature architecture, limb orientation, and skeletal stresses and strains [2,3,4,15]. During walks and runs, birds’ limb bones are subjected both to torsional stresses and compressive stresses alongside the proximo-distal axis of the bone, directed with respect to the proximo-distally axis, due to the action of the musculature and the change of orientation during the movement [4]. Both femora and tibiae are subjected to torsional and bending stresses (Figure 1), with the femoral heads and the distal portion of the tibiae rotated medially, while the knee, affecting the distal portion of the femora and the proximal portion of the tibiae, is subjected to a rotation directed laterally [4]. Due to the orientation of bones during motion, the main axis of compression forces is oriented at around 25° with respect to the proximo-distal axis of the tibiae. In comparison, the femoral main axis of compression is angled at around 37–49° with respect to the proximo-distal axis [4]. The magnitude of stresses is directly dependent on the body mass, the accelerations applied, and changes during growth, yet the orientation and proportions are maintained constant during the lifetime of the animal, not being affected by ontogenetic processes [4]. Thanks to the wide documentation on ostrich anatomy, physiology, and dynamics of motion, it is an ideal case for studying the adaptations of bone structures to sustain stresses.

#### 1.1.1. Bone Structure

Bones in any animal skeleton must fulfil different physiological roles, with the main ones being the structural support for the entire body, the protection of delicate internal organs, and the structural support to withstand the loads and accelerations imposed by the movements. Bones are organs constituted by living tissues adapting to external stimuli, able to remodel and adjust the bone after traumas, such as a fracture, or even adapting to the removal of a significant portion of tissues [16,17,18]. Bone, as a material, can be differentiated into the cortical bone (also known as compact bone), having a porosity of 5–15%, and the spongeous bone (also known as trabecular bone or cancellous bone), having a porosity of 40–95% [19]. Long bones are commonly organized as having the spongeous bone concentrated at the epiphyses, while the compact bone is concentrated in the diaphysis [20]. This distribution of tissues within the bone reflects different mechanical properties, with spongeous bone having a lower strength and Young’s modulus (E) than compact bone [14]. It has been hypothesised that the orientation of the trabeculae composing the spongeous reticulum corresponds to the direction of strains acting on the bone and that spongeous bone plays a role in energy dissipation, though these arguments are debated [21]. Bones at the microscopic level are composed of the protein collagen organized in fibres, with these fibres embedded in an amorphous matrix mainly composed of calcium phosphate, and with an important percentage of water that affects the mechanical properties [21]. Multiple factors influence the mechanical strength of bones, such as the main orientation of the collagen fibres, the species of the animal, its age and sex, the density and degree of crystallization of the bone, its position within the body, and the conditions under which the tests are conducted, such as the moisture of the samples and the strain rate at which the loads are applied during testing [14,22,23,24,25]. Bone has an orthotropic behaviour conferred by the orientation of the collagen fibres within the bone tissue, an orientation that is parallel to the long axis of the diaphyses [26]. The orientation of the collagen fibres is obtained through the process of ossification during growth, adapting to the external loads. Although the magnitude of loads varies with age, the orientation and distribution remain constant during an individual’s life [4].

**Figure 2:**
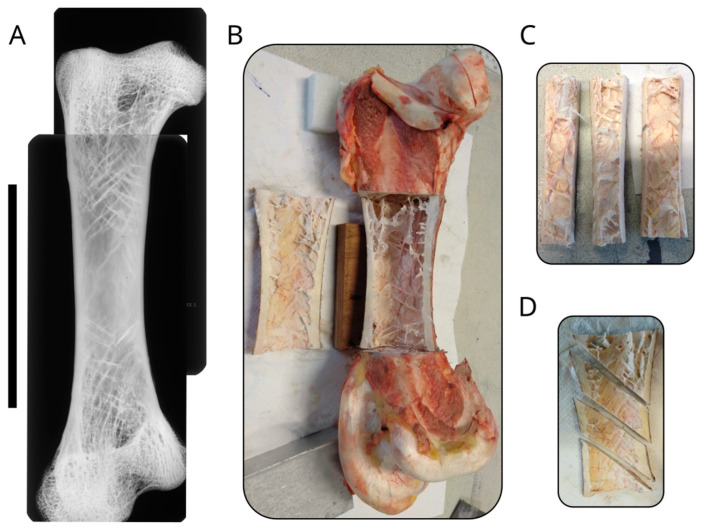
Structure of the ostrich femur. (**A**) Image composed of multiple radiographic images of an ostrich femur, showing the distribution of the trabeculae. The scale bar is equal to 10 cm. (**B**) Photo of the sectioned femur showing the marrow cavity with the oriented trabeculae. (**C**) Photo of the samples oriented alongside the long axis of the femur. (**D**) Photo of the samples oriented at 45° with respect to the long axis of the bone.

Bones are subjected to strict geometric constraints, and their shape has evolved to accommodate the loadings to which they are subjected. The enlarged extremities of bones have the role of increasing the area of contact within the articulations, thus reducing the local pressure applied at the contact between different bones. The loadings are accommodated by a change in the shape and in the material of the bone, with the trabeculae net of the spongeous bone arranged at the epiphyses, which is useful to redistribute the loadings from the articular facets to the shaft, mainly composed of cortical bone, as can be observed in the radiographic images of the ostrich femur (Figure 2). The stresses acting on the bone shaft are independent of the type or distribution of the load applied, since the stresses are distributed homogeneously on the main shaft’s section, due to the principle of de Saint-Venant [27,28].

**Figure 3:**
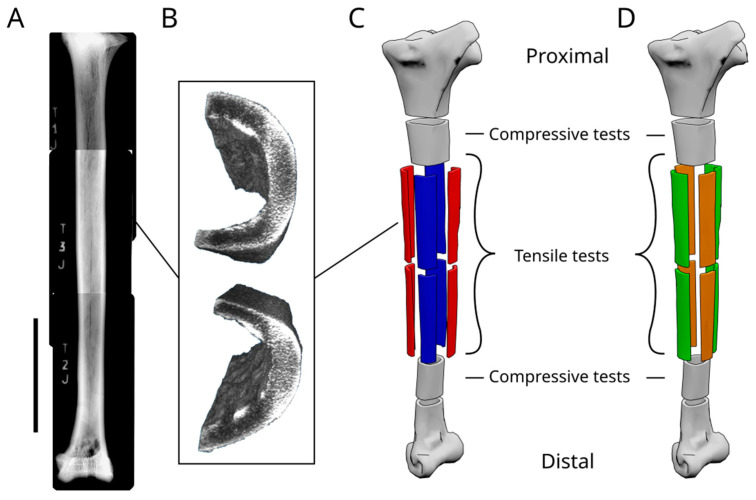
Structure of the ostrich tibia. (**A**) Image composed of multiple radiographic images of an ostrich tibia, showing the internal structure. The scale bar is equal to 10 cm. (**B**) Image obtained from the CTscan data showing the differences in density within the tibial shaft. (**C**) Model of an ostrich tibia showing how the samples are obtained from the dried tibia. (**D**) Model of an ostrich tibia showing how the samples are obtained to perform the mechanical tests.

The femur is stout and short compared to the tibia, a typical adaptation for animals to run. The femur has an intricate system of bone trabeculae filling the medullar cavity, mainly concentrated near the epiphyses of the bone, constituting a stiffer structure to support the stresses applied on the femoral head and at the articulation with the knee. The intricate system of trabeculae unravels towards the mid-shaft, where the trabeculae are oriented at 45° with respect to the proximo-distal axis of the bone and are concentrated towards the perimetral wall, fusing with it, creating bone ridges (Figure 2). The tibia is slender and longer than the femur, with the spongeous bone constituted by a thick net of bone trabeculae, concentrated at the epiphyses of the bone, while the marrow occupies the central portion of the medullar cavity. The thickness of the peripheral walls varies along the long axis of the bone, with the maximum thickness found at the distal end of the shaft, where the peripheral circumference is at its minimum (Figure 3). Such adaptation, of having a thicker wall with a lesser circumference is an adaptation to increase the stiffness of the structure, to maintain the same performances as in other portions of the bone.

#### 1.1.2. Density

The act of walking generates torsional and bending forces on the bones, and this creates cyclical stresses that are directed differently in different regions of the same bone. Multiple samples have been obtained from the same bone in order to test material adaptations in the different regions of the same bone. Samples for similar tests were kept under the same conditions before and while performing the tests. While testing, two parameters emerged as critical in interpreting the results, and thus have been deepened: the density of the bone tissues and the moisture of the samples. The density of cortical bone is around 2000 kg/m [3], and it slightly varies during the lifetime of an individual as it is affected by health conditions, such as ontogenetic development or diseases [1,2,29,30]. In humans, the density of bones increases until reaching the apex at around 30 years, remaining constant for around ten years, then decreasing for the rest of their life [30]. Birds have a rapid growth rate and a consequent rapid increase in bone density [2,31], and to maintain the necessary strength required by bones, while reducing their weight, birds have dense bones compared to mammals [32]. Density has a significant role in the mechanical properties of the bone, both in bone mineral density and in the porosity of the bone [33]. Different approaches were applied in this study to estimate the density of bones and measure the effects of bone density on mechanical properties: the ostrich bones were radiographed and CT-scanned, and samples were weighted with Archimedes’ method to compare the results obtained from the mechanical tests. Only Archimedes’ method was used to obtain numerical results, and tests were carried out using a Mohr’s balance. The degree of porosity is also related to the degree of saturation of bone, with water being a principal component of bone [34]. Water represents in human bones up to 25% of the volume of cortical bones [35]. The moisture of the sample affects the material properties; in fact, in saturated bones, the stiffness is increased compared to dried bones [36]. A series of measurements was carried out to calculate the saturation curve and extrapolate the samples’ moisture level at the moment of the tests.

#### 1.1.3. Isotropy and Orthotropy

The isotropic material is a material that possesses equal properties in response to external stimuli uniformly in all directions, from the Greek for “equal” and “way”. The orthotropic material has different material properties, depending on its orientation. To obtain the orthotropic properties, the material usually has to be a composite material. Composite material has two components: a homogeneous and compliant matrix, and stiff fibres embedded in the matrix and oriented in a single direction. The orientation of the fibres grants to the composite material the properties of the stiffer component to the whole material when tested parallel to the fibres, while when tested orthogonally to the fibres, the material acquires the properties of the matrix. Bone is often compared to composite materials having stiffer collagen fibres immersed in the amorphic matrix. To obtain the constitutive law of an isotropic material, a simple tensile test provides the necessary data: the Young’s Modulus (E) and the Poisson ratio (𝜈). In order to create a constitutive law for an orthotropic material, such as bone, it is required to measure Young’s Modulus along the main axis of resistance (E_x_) and along the lesser axis of resistance (E_y_), as well as the Shear Modulus (G) and the Poisson ratio. In limb bones, the main axis of resistance corresponds to the main axis of the bone, along which are concentrated the major stresses. The easiest way to obtain the E_x_ is by performing uniaxial tensile tests on samples from the tibial shaft. Measuring the E_y_ requires a different approach, since the bone is too curved to obtain classical uniaxial tensile samples; thus, cylindrical samples were obtained and tested with the O-ring technique. G was estimated from rectangular samples extracted from the tibial shaft, maintaining the direction of load parallel to the original long axis of the bone.

### 1.2. Finite Element Method

The increased capability to create 3D models of extant objects has made the use of computational methods, such as Finite Element Analysis (FEA), easily accessible, rendering them a popular tool in many fields of science, including palaeontology [37]. In particular, FEA is becoming more and more popular, as a useful tool for analyses and testing hypotheses without the risk of damaging the actual specimens and providing results unattainable before its implementation. FEA requires accurate geometry of the 3D objects and accurate material properties. While obtaining the geometry of an object has seen significant development, with the increased popularity of accurate instruments such as computer tomography (CT), laser scanning, and photogrammetry [38], often the model used for the simulation of bone material properties is homogeneous and isotropic [6,39,40,41,42]. Choosing a homogeneous isotropic bone model is of great aid in reducing computational time and is useful for comparing models through 3D geometry [39,40]. However, the use of anisotropic models has given better results than isotropic ones [43], and bone is known to be an orthotropic material and to have unevenly distributed density [2]. The accuracy of the bone material model is of fundamental importance, since FEAs can provide results useful in medicine and veterinary ambits. The results of FEA performed using modern specimens can be validated by data obtained ex vivo on patients, a fact that puts palaeontological studies at a disadvantage, since a modern equivalent often does not exist. It is common practice to use a generalised E of bones in FEA paleontological studies, mainly extrapolating the data from studies performed on different animals, such as humans and cattle, the most commonly studied, with data principally obtained from three-point bending tests. However, these data cannot be properly used for other animals’ FEAs due to the level of inaccuracy they bring to the simulation. Data obtained from literature usually exaggerate E values, primarily while considering mammal bones in studies devoted to reptiles or birds. The three-point bending test is also unreliable due to the bone shape and structure, thus affecting the test results. In fact, the entire bone is solicited during the three-point bending test; this way, the test provides results relative to the entire bone structure and not the actual mechanical material properties of the bone, most of the time overestimating the bone E. Another factor overlooked in the three-point bending tests is the regionalization of the bone; in fact, during the test, the different regions would accommodate the load deforming in different ways, redistributing the stress within the entire structure. As presented in this study, the mechanical properties of bone, as a material, also vary within the same bone; thus, multiple samples should be obtained from each bone tested to sample the different bone regions. Due to the orthotropic nature of bone material, the main direction of loads applied in life should be considered when comparing samples from a single bone, since a sample aligned along the main axis of the bone may not be aligned to the main axis of loading in life. The bone, being a living organ, will adapt its shape and structure in life to sustain the daily loads at its best; omitting all these factors (the animal’s age and lifestyle, the typical orientation of the bone in life, the direction of loads, etc.) during the mechanical tests will lead to false E values, thus affecting the simulations. A generic homogeneous bone model, with a generic E, can still be helpful in comparing different bone morphologies, which can be useful for palaeontological specimens. However, these simulations should be considered informative about the distribution of the stress within the structure tested, but not entirely informative on the ultimate loads bearable by such structures. Still, as a general rule, it is recommended to use data obtained from the closest relative of the animal studied, using the best available data for cautious application to new studies.

## 2. Materials and Methods

### 2.1. Materials

#### 2.1.1. Origin

The materials used in this study consisted of a tibia, donated by the MSNM (Museo di Scienze Naturali di Milano), and one femur and two tibiae, acquired from a commercial butcher. The material donated by MSNM is of unknown age and was kept in the collection with no data on its preservation or date of acquisition from the museum. The inner structure of the tibial bone presented remodelled tissues, with at least five bone rings, a clear indication that the animal, at the time of death, was an adult of at least five years old [31,44] (Castanet et al., 2000; Horner et al., 2000). The tibia was kept under environmental conditions. The material purchased by the butcher belonged to a young individual, bred in a commercial ostrich farm, of an age of approximately 9–18 months, the typical age when the animal reaches 100 kg of weight and is ready to be processed for the culinary industry. The bones were kept in a refrigerator at −18 °C until processing. Animal handling norms were respected, and no harm was caused to any animal for the purpose of this study, using material obtained as waste from the commercial culinary industry.

#### 2.1.2. Density

The bone density was measured using a Mohr-Westphal hydrostatic balance; two samples, one from the craniocaudal portion and one from the lateral portion of the young tibia, were obtained from previously tested tensile samples. The samples were left to sit drying under environmental conditions before being immersed in distilled water and measured.

The radiographic machine housed at Politecnico di Milano was set to run with a power of 50 kV at 6 mA for an approximate time of 55 s, in order to obtain a clearer definition.

A CT-scan of the tibial shaft and the femoral shaft were obtained using an AMALA NSI-X25 Computed Tomography System, with a resolution of 1340 × 524 × 1849 voxels, with each voxel corresponding to 61.67 × 61.67 × 61.67 microns for the tibia, and a resolution of 1292 × 1503 × 1001 voxels, with each voxel corresponding to 49.91 × 49.91 × 49.91 microns for the femur.

#### 2.1.3. Mechanical Characterization

**Figure 4:**
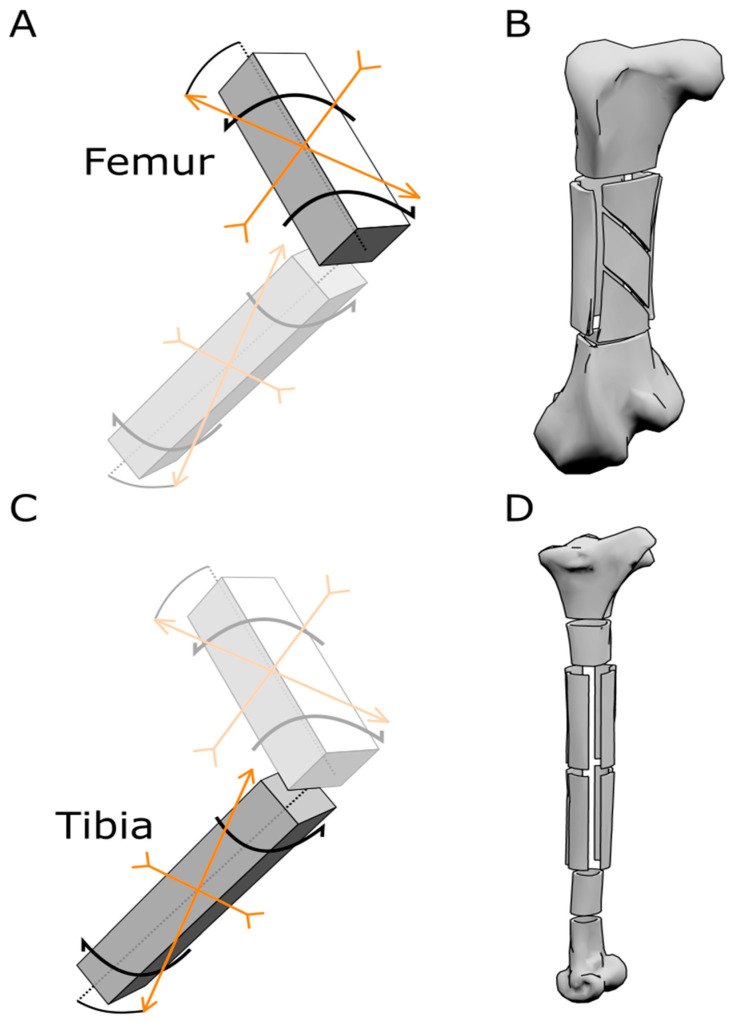
Scheme of force distribution and samples obtained from the ostrich bones. (**A**) Schematic model representing the direction of forces acting on the femur. (**B**) Model of an ostrich femur representing how the samples for the mechanical tests were cut out of the bone. (**C**) Schematic model representing the direction of forces acting on the tibia. (**D**) Model of an ostrich tibia representing how the samples for the mechanical tests were obtained from the bone.

The Young’s modulus along the main axis (E_x_) was valued using unidirectional tensile strength tests of the bone samples. Eight rectangular samples were cut from the tibial shaft, with a length of 130 mm and a width of around 20 mm, depending on the shape of the diaphyses, maintaining the thickness of the bone; four samples from the proximal tibial shaft, and four samples from the distal tibial shaft (Figure 4). The extremities of the samples were embedded in bi-component resin mixed with aluminium powder in order to create a grip zone to guarantee a better hold by the tensile testing machine and protect the samples from the pressure applied by the jigs. These resin extremities were milled to be adapted to the dimensions of the tensile test machine (Figures S1 and S2). To evaluate the E_y_, a different approach was used. Due to the natural shape of the tibial shaft, having a nearly cylindrical section, the tests were conducted using O-ring samples. Cylindrical samples were obtained from the tibial shaft extremities. The marrow was removed, and the medullar cavity was filled with bicomponent resin mixed with aluminium powder, leaving an interstice of millimetric thickness in order to direct the forces only on the bone and not on the resin. Steel pins of 6 mm in diameter were inserted in the resin moulds within the medullar cavity of the samples and used to apply the forces needed to carry out the tests. The pins were connected to the testing machine by two forks. G was measured using single shearing tests; rectangular samples were obtained from sections of the diaphyses and tested by imposing compressive stress on a side directed parallel to the original long axis of the bone, while holding still the other side of the sample. All mechanical tests were conducted registering data every 0.1 s, with a load velocity of 0.5 mm/min, using a servohydraulic testing machine in force control (Figures S3–S14). Young’s modulus was calculated in Matlab (Matlab Simulink) using the curve-fit linear proportion.

#### 2.1.4. Water Absorption

The water absorption of bones was measured by immersing the bone samples in physiological liquid, keeping the temperature stable at 37 °C using a thermal bath. The samples were weighed at regular intervals for four days using a precision scale up to one-thousandth of a gram. Seven samples were destined for the saturation tests: five samples were obtained from different regions of the tibial shaft, representing both the craniocaudal sides and the mediolateral sides, and two samples were obtained from the tibia donated by the MSNM; this bone was kept dried for years. These samples were cut and kept under environmental conditions for 48 h before beginning the test.

## 3. Results

### 3.1. The Density of the Bone

The CT scan of the tibial shaft highlighted a regionalization of the shaft, in quadrants having different densities. The regions with denser bone tissue were the lateral and proximal ones, while the cranial and caudal faces of the tibial shaft were less dense. Two Mohr-Westphal hydrostatic balance measures were carried out six months apart. The first measurement resulted in the lateral sample having a density of 2068 kg/m [3] and the craniocaudal sample having a density of 1958 kg/m [3]. The second measurement was carried out six months later; the lateral sample had a density of 2068 kg/m [3], and the craniocaudal sample had a density of 1945 kg/m [3]. 

### 3.2. Mechanical Characterisation of Ostrich Bone

**Figure 5:**
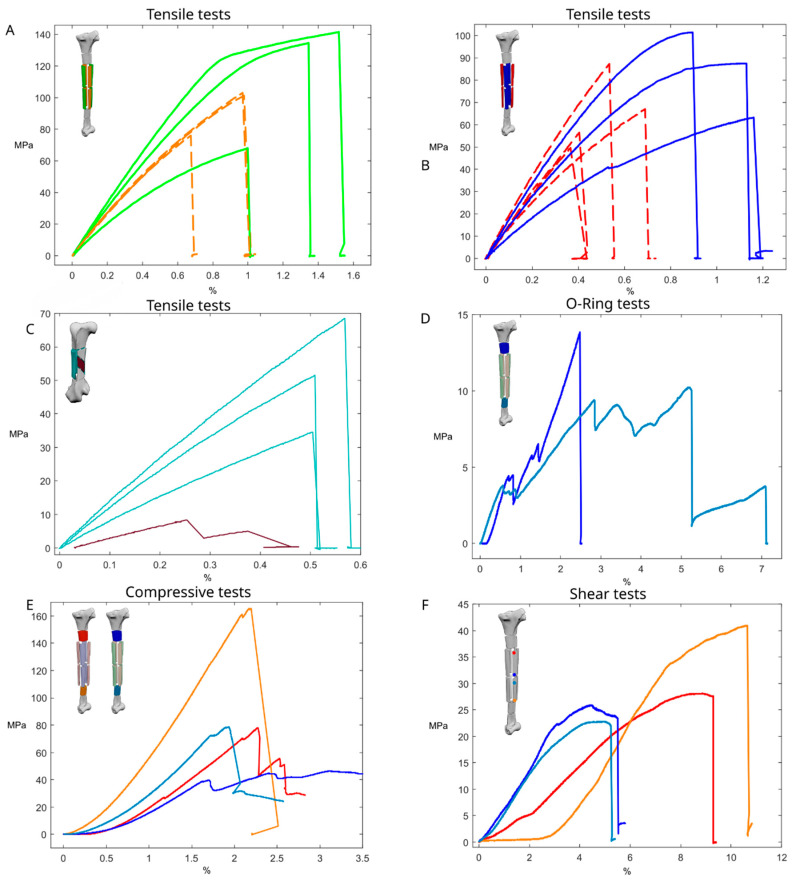
Mechanical tests. (**A**) Stress–strain curve of the tensile tests on the lateromedial regions of the tibial shaft (full line, green); stress–strain curve of the tensile tests on the frontal-caudal regions of the tibial shaft (dashed line, orange). (**B**) Stress–strain curve of the tensile tests on the wetted samples of the tibial shaft (full line, blue); stress–strain curve of the tensile tests on the dried-out samples of the tibial shaft (dashed line, red). (**C**) Stress–strain curves of the samples obtained from the tibial shaft; in red the proximal region of the shaft, in blue the frontal mid-shaft sample, in light blue the lateral mid-shaft sample, and in orange the distal shaft sample. (**D**) Stress–strain curve of the O-ring samples obtained from the ostrich tibia; in dark blue the proximal region, and in light blue the distal region. (**E**) Stress–strain curves of the compression tests of samples from the ostrich tibia; in dark blue the wetted proximal region, in light blue the wetted distal region, in red the dried proximal region, in orange the dried distal region. (**F**) Stress–strain curve of the tensile tests of samples obtained from the femur, samples aligned to the long axis of the femur (full line, light blue); stress–strain curve of the tensile tests on the sample aligned at 45° with respect to the long axis of the femur (full line, brown).

Tensile tests along the major axis of the bone were performed on the craniocaudal samples, highlighting a brittle behaviour with an ultimate tensile strength (σ_u_) of values ranging from 75.99 MPa to 103.02 MPa, with an E_x_ ranging between 12.53 GPa and 14.6 GPa. The tests performed on the lateral and medial sides of the tibial shaft highlighted a distinct elastoplastic behaviour, with a yielding stress (σ_Y_) of ≅120 MPa, an σ_u_ of 134.55 MPa and 141.49 MPa, and an E_x_ ranging between 15.3 GPa and 17.3 GPa (Figure 5, Table 1).

Tensile tests perpendicular to the major axis of the bone were carried out using the O-ring test procedure, resulting in σ_u_ ranging between 9.37 MPa and 13.85 MPa and E_y_ ranging between 0.07 GPa and 0.11 GPa (Figure 5).

The shear tests conducted on the sample from the proximal region of the tibial shaft resulted in a maximum shear stress (τ_u_) of 27.54 MPa and a shear modulus (G) of 0.4 GPa. The samples from the central region of the shaft evidenced a τ_u_ of 23.9 MPa and 22.77 MPa, with a respective G of 0.9 GPa and 0.7 GPa. The sample from the distal region of the tibial shaft evidenced a τ_u_ of 40.92 MPa and a G of 0.8 GPa (Figure 5).

The compressive tests conducted on tibial samples were conducted on dried samples, resulting in a σ_u_ ranging between 78.01 MPa and 165.4 MPa, with an E_x_ ranging between 4.7 GPa and 10.4 GPa (Figure 5), and on saturated samples resulting in a σ_u_ ranging between 39.6 MPa and 78.56 MPa, with an E_x_ ranging between 3.8 GPa and 6.58 GPa (Figure 5), with the samples from the distal portion resulting in having the best performances.

The tensile tests conducted on the femur with samples aligned with the long axis of the bone resulted in a σ_u_ ranging between 34.59 MPa and 68.56 MPa, with an E_x_ ranging between 7.51 GPa and 13.2 GPa (Figure 5). The test conducted with the sample oriented alongside the internal ridges resulted in a σ_u_ of 8.25 MPa and E_y_ of 3.75 GPa (Figure 5, Table 1).

### 3.3. Saturation Results

**Figure 6:**
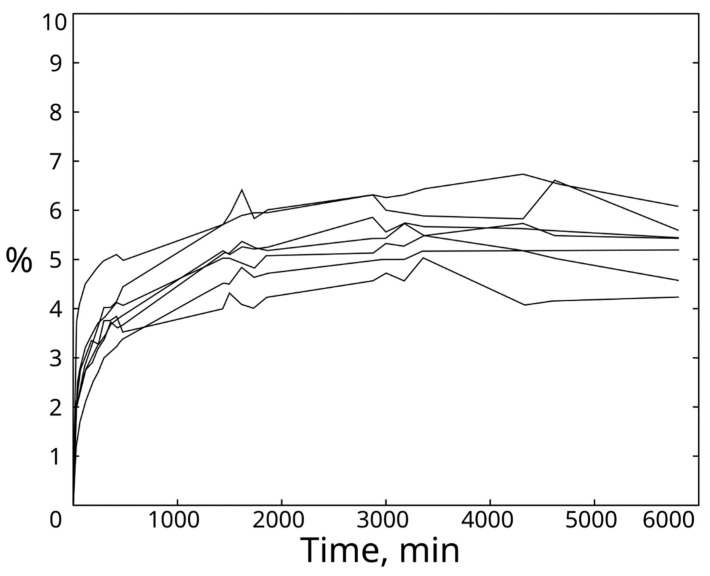
Curves of water absorption. Curves were obtained by plotting the data obtained from the seven samples tested to measure the water absorption of ostrich bones.

Due to the great porosity of bones, the measurements taken highlighted a rapid absorption of water in the early hours of the experiment, reaching around 80% saturation within 6 h of the beginning of the test. The increase in mass of the samples was recorded to be around 5% of the initial mass of the sample, reaching the asymptote within 10 h from the beginning of the test. The seven samples showed no particular difference in water saturation, even if belonging to different portions of the same bone (Figure 6).

### 3.4. Change in Mechanical Properties Due to Saturation

Three samples were obtained from the dried-out tibia and were tested after keeping them under environmental conditions. These samples evidenced a brittle behaviour, reaching a σ_u_ value between 49.63 MPa and 87.33 MPa and having an E_x_ ranging between 12 GPa and 18.5 GPa (Figure 5). 

Three samples were kept immersed in water under environmental conditions for one night before testing, reaching an acceptable level of water saturation for the material (more than 80%). These samples had a stiffer and tougher behaviour compared to the dried samples, with a gentle and progressive change from elastic to plastic behaviour, also achieving higher E_x_ and σ_u_ values. The σ_u_ varied between 63.23 MPa and 101.35 MPa, with an E_x_ value ranging between 8.24 GPa and 16.08 GPa (Figure 5).

## 4. Discussion

### 4.1. Mechanical Testing and Implications for Literature Data

#### 4.1.1. Femur

The ostrich is the fastest bipedal animal alive today, due to the adaptations of the limb bones. The differences between the femur and the tibia reflect the optimization of such structures to sustain the stresses imposed by running at a fast speed for a long period (Figure 1). The femur is articulated with the pelvis at an angle with respect to the dorsoventral and craniocaudal axes. Such orientation affects the distribution of forces generated by the musculature connecting the femur to the rest of the body, with a resultant incline in a range of 37–49° with the proximo-distal axis of the bone, thus creating a strong torsional component acting on the bone during each stride, with a lateromedial torsion working at the proximal end of the femur and a mediolateral torsion working at the distal end of the femur during the extension of the limb [4] (Figure 4). To sustain such stresses, the shape of the ostrich femur has evolved into a short and stout bone, acting as an advantageous lever during the swing phase, and with a stiff structure as evidenced by the distribution of the internal trabeculae, oriented to maximise the distribution of stresses. These trabeculae form the spongeous bone near the epiphyses and, in the medullar cavity, form a complex structure of intersecting bone ridges, inclined at 45° with respect to the long axis of the bone (Figure 2). Similar solutions are commonly exploited in different engineering applications to resist torsional stresses [44]. The bone ridges present in the medullar cavity of the femur act naturally as reinforcements to support the tensions and strengthen the structure to support the bending stresses imposed on the bone during the walking and running phases.

#### 4.1.2. Tibia

The tibia is responsible for sustaining the compression stresses acting on it during the stance phase, while the foot is touching the ground and the full body mass is sustained by the only leg in contact with the ground. The tibia is longer and more slender than the femur, and its position and orientation allow the applied forces to be directed at 25° with respect to the long axis of the bone, with the tibia only slightly tilted on the craniocaudal axis, and the knee in a more lateral position than the ankle (Figure 1) [4,11]. This inclination is reflected by the shape, structure, and material properties of the tibia itself. Since the tibia is posed laterally to the centre of the mass of the body, it is constantly stressed by a flexural momentum; this condition is reflected by the different mechanical behaviour observed in the different samples tested and in the distribution of the density. The presence of less dense regions in the tibial shaft may be attributed to the immaturity of the individual, and further studies may highlight the variation of bone density during ostrich growth. However, the results of the mechanical tests and the density are correlated, with the denser samples being stiffer and tougher and having higher σ_u_ and E values than the less dense samples. The distribution of the denser regions to the lateral and medial sides of the tibial shafts reflects the torsional momentum acting on the bone and the distribution of loadings acting on the bone during the walking/running cycle. The tensile tests carried out on the samples oriented alongside the main direction of loadings highlight a clear orthotropic behaviour of the bone. The results obtained from the mechanical tests confirm the literature data, with E ≅ 17 GPa in tensile tests aligned to the long axis of the bone [7,12,14]. The E_x_, directed perpendicular to the long axis of the bone, is 100 times smaller than the E_y_, directed parallel to the long axis of the bone, with values of around 0.1 GPa, highlighting a clear orthotropic behaviour, with the most bearing axis coincident with the main axis of loading of the bone. Previous studies conducted on estimating the radial E of bones performed their tests using the three-point bending test; however, due to the nature of the test, the entire bone structure is solicited. At the same time, the O-Ring technique is susceptible to influence by multiple factors, with the main factors related to environmental conditions, as creating the resin supports requires time to consolidate the resin itself. In this regard, further tests should be performed to better comprehend the degree of orthotropy of bone as a material, avoiding testing it as a structure.

The shear stress values obtained from the ostrich tibia (0.8 GPa) are comparable to previous studies, which report a general shear stress of 1 GPa both for birds and mammals, with no differences reported from the different animal groups [12]; yet, the ratios E/G results are inferior to previously reported values [12]. The shear values obtained during the experimental tests presented in this study and the ones presented by Spatz et al. [12] are inferior to the values reported by Gilbert et al., [2] who reported values in the range from 3.65 to 5.1 GPa [2]. This difference can be attributed to the use of values obtained from tests conducted on human bones [45], as proposed by Gilbert et al., due to the lack of data for ratites [2].

Bone is a porous material, mainly due to the canals present within the structure itself. Even when completely dried, bones reach saturation in 8–10 h after immersion, with 60% of saturation reached within the first 30 min of immersion. Water-saturated bones change their mechanical behaviours, from a brittle-elastic behaviour to a more plastic and yielding behaviour. Similar observations were already proposed in the literature, though these were related to compression tests, where it was hypothesised that water takes part in the stress resistance of the material. During these tests, it was observed that water-saturated samples were better performing than dried ones, with performances similar to ex vivo conditions. Hence, the moisture conditions of bone samples highly affect the results obtained from mechanical tests.

#### 4.1.3. Engineering and Nature

Engineering and nature face similar issues, and solutions to problems are developed through research and evolution. Examples are Greek and Roman columns, where the capital has a larger surface in contact with the overlaying beam; to reduce the local pressure applied to the capital; a similar solution is adopted in nature, with enlarging the epiphyses of long bones. Columns and bones follow the principle of de Saint-Venant, reducing their diameter and adopting a semi-circular section, useful for maximising the section area and reducing the external surface. Another example is the geodetic structure, developed to counteract the torsional momenta acting on said structure. An example is the airframe of the fuselage and wings of the English Vickers Wellington, where the geodetic structure was designed to withstand the torsional momenta acting on the aircraft. The same structure can be observed in the arrangement of the ridges in the marrow cavity of the ostrich femur, a solution useful to strengthen the torsional resistance of the bone. Up to now, metallurgical processes have not reached the capability to produce alloys with different densities within a single structure, unlike the ostrich bone, where the regionalization of the tibial shaft coincides with the most prominent bending momenta. To address this deficiency, great care is posed in designing the most optimised shape, a process parallel to evolution, where the most suitable structure is the favoured one. Nature and engineering face similar constraints as well; among them, there are geometric and material limitations. Due to limitations of the metabolic processes, nature is unable to produce any alloy and thus is limited to synthesising organic materials, while engineering has access to a wider array of materials but lacks the capability of regenerating damage or producing a single alloy with variable density and mechanical properties.

## 5. Conclusions

The ostrich bones analysed in this study show stress-bearing adaptations of great interest, which likely are present in other animals as well. Bones have a clear orthotropic behaviour, with the most-bearing axis corresponding to the main axis of loading forces, which does not always coincide with the long axis of the bone. As a general rule, avian bones have a smaller Young’s modulus than mammals, with the most-bearing axis having a Young’s modulus of around 17 GPa. Further studies are required to better estimate the radial Young’s modulus of bone and the shear modulus. The porosity grants the capacity to reach water saturation in a small amount of time: 60% within 30 min since immersion. These results are mainly due to the structure of bones, having canals of different sizes that are present even in the so-called compact bone. The moisture of bones greatly affects their material properties, with a moist material being more yielding and resistant to stress than a dried one, not only in compression tests but in tensile tests as well.

Bones are complex structures composed of orthotropic bone material, which is suitable to sustain loadings that typically occur during the lifetime of an individual. Both the bone structure and the bone material are adaptable to the necessity imposed by the organism itself, with the capacity of adapting to environmental conditions. The “strengthened” regions of the ostrich tibial shaft show that the denser quadrants correspond to the regions where the loadings are concentrated and correspond to the best-performing material qualities. This selective regionalization is of high interest, since the bone assumes the contours of a smart structure, able to adapt to the external loading conditions not only by changing its shape but also by modifying its own material properties. The capacity to adapt the bone shape to best exploit its use is a known topic in science and one of the fundamental aspects of anatomy; however, the capacity to selectively modify the material property is here firstly described, opening a new window of research on bone materials as a smart structure composed by a smart material.

The results obtained by this research may spark interest in engineering in terms of the capacity of nature to produce a material that is self-adaptable and can adjust to the loadings applied to it. Moreover, other solutions may be found by studying other animals; thus, biomimetic research should be incentivized to find more optimized and efficient structures and develop technologies and procedures to replicate them.

## Figures and Tables

**Table 1:** Table illustrating the results of the tests conducted on ostrich limb bones.

	E_x_ GPa	σ_u_ MPa		E_y_ GPa	σ_u_ MPa	G GPa		Τ_u_ MPa
Tibial Shaft Proximal Portion	15.3	134.55		0.7	13.85	0.8		27.54
Tibial Shaft Distal Portion	17.3	141.49		0.11	9.37	0.4		40.92
	**E along main axis bone** **GPa**		**σ_u_ along main axis bone** **MPa**		**E parallel to ridges orientation** **GPa**		**σ_u_ parallel to ridges orientation** **MPa**	
Femur	13.2		68.56		3.75		8.25	

## Data Availability

Not applicable.

## Supplementary Materials

The following supporting information can be downloaded at: https://www.mdpi.com/article/10.3390/biomimetics8010098/s1, Figure S1: Tibia dried samples; Figure S2: Tibia wetted samples; Figure S3: End of uniaxial tensile test; Figure S4: Sample 4.01; Figure S5: Sample 4.02; Figure S6: Sample 4.03; Figure S7: Sample 4.04; Figure S8: Sample 6.01; Figure S9: Sample 6.02; Figure S10: Sample 6.03; Figure S11: Sample 6.04; Figure S12: Sample 6.05; Figure S13: Sample 6.06; Figure S14: Sample 6.07.
