# Supplementary material for: Smart Biomechanical Adaptation Revealed by the Structure of Ostrich Limb Bones
Source: Biomimetics (Basel). 2023 Feb 28;8(1):98. doi: 10.3390/biomimetics8010098 (PMC10046004; doi:10.3390/biomimetics8010098)
Supplement: Supplementary file 1 [file biomimetics-08-00098-s001.zip › biomimetics-2215084-supplementary.pdf]

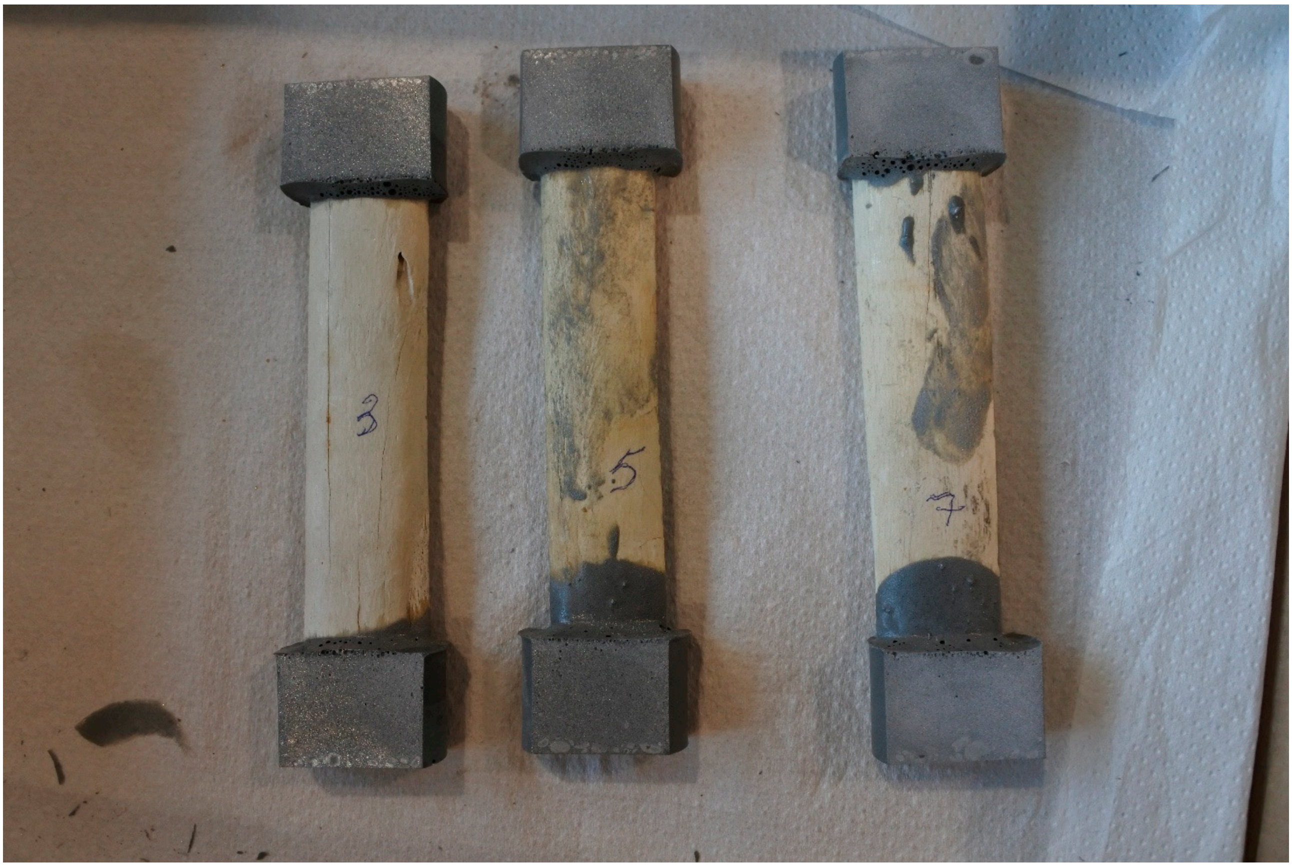

Figure S1: Tibia dried samples. Dried samples ready for uniaxial tensile test.

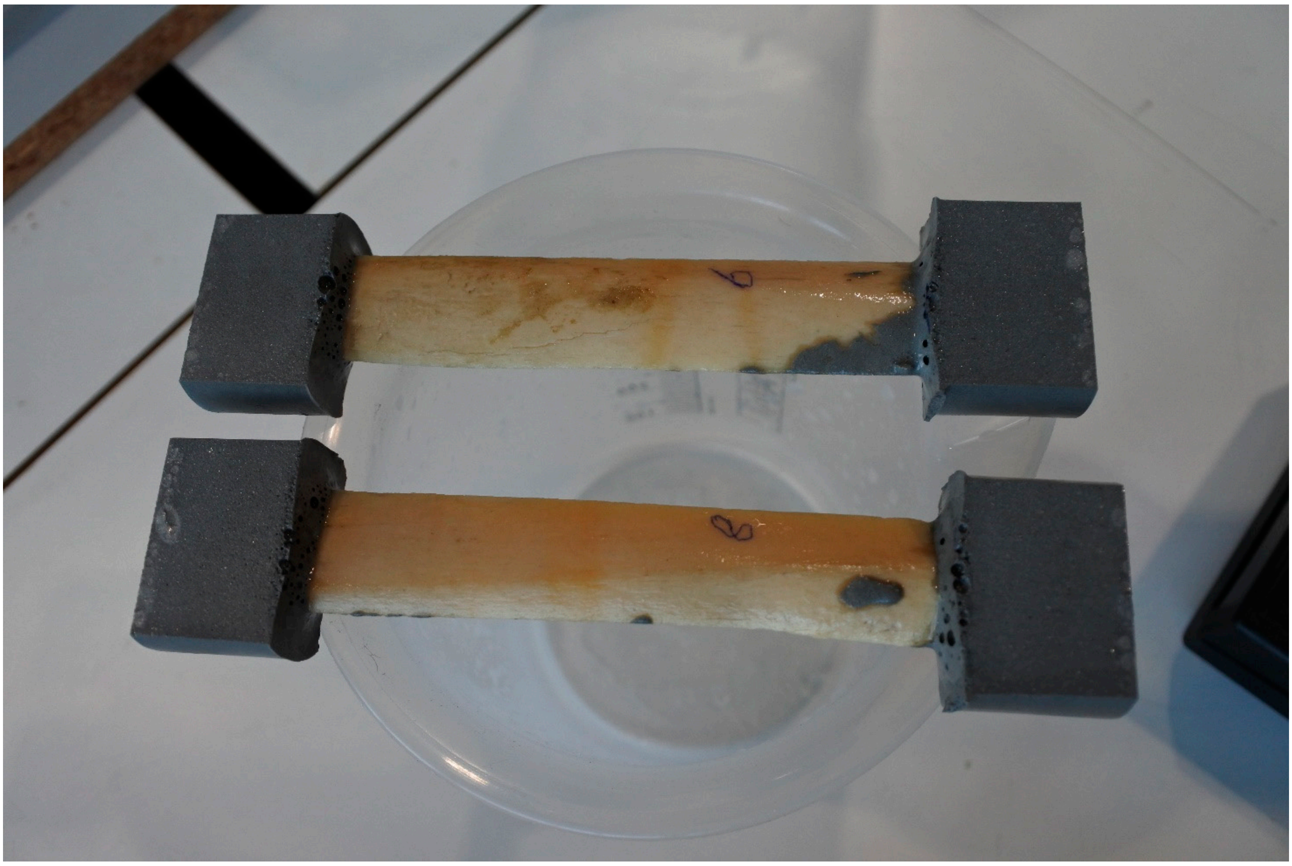

Figure S2: Tibia wetted samples. Wetted samples ready for the uniaxial tensile test.

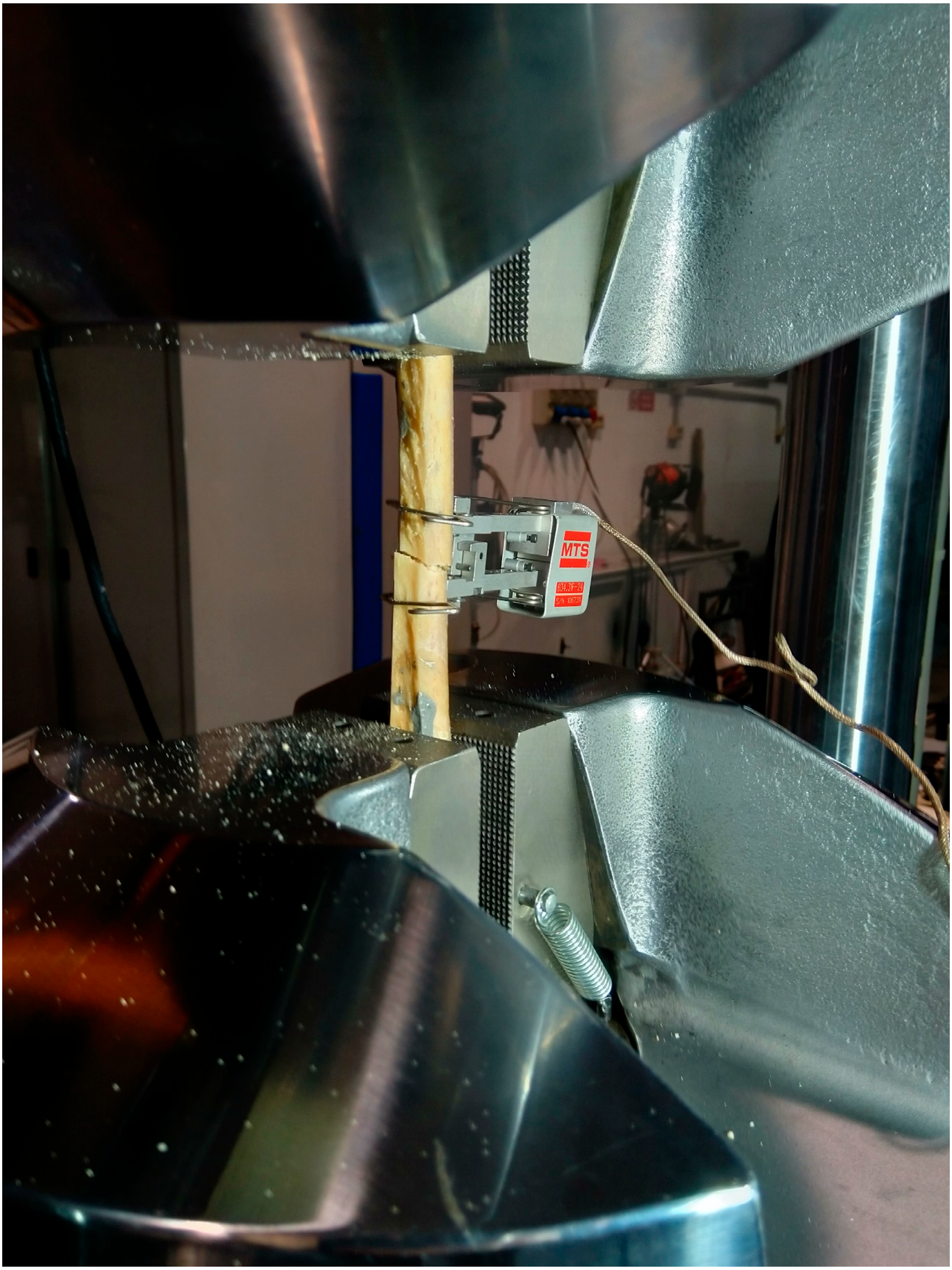

Figure S3: End of uniaxial tensile test. Final stage of the uniaxial tensile test conducted on a sample of the tibial shaft.

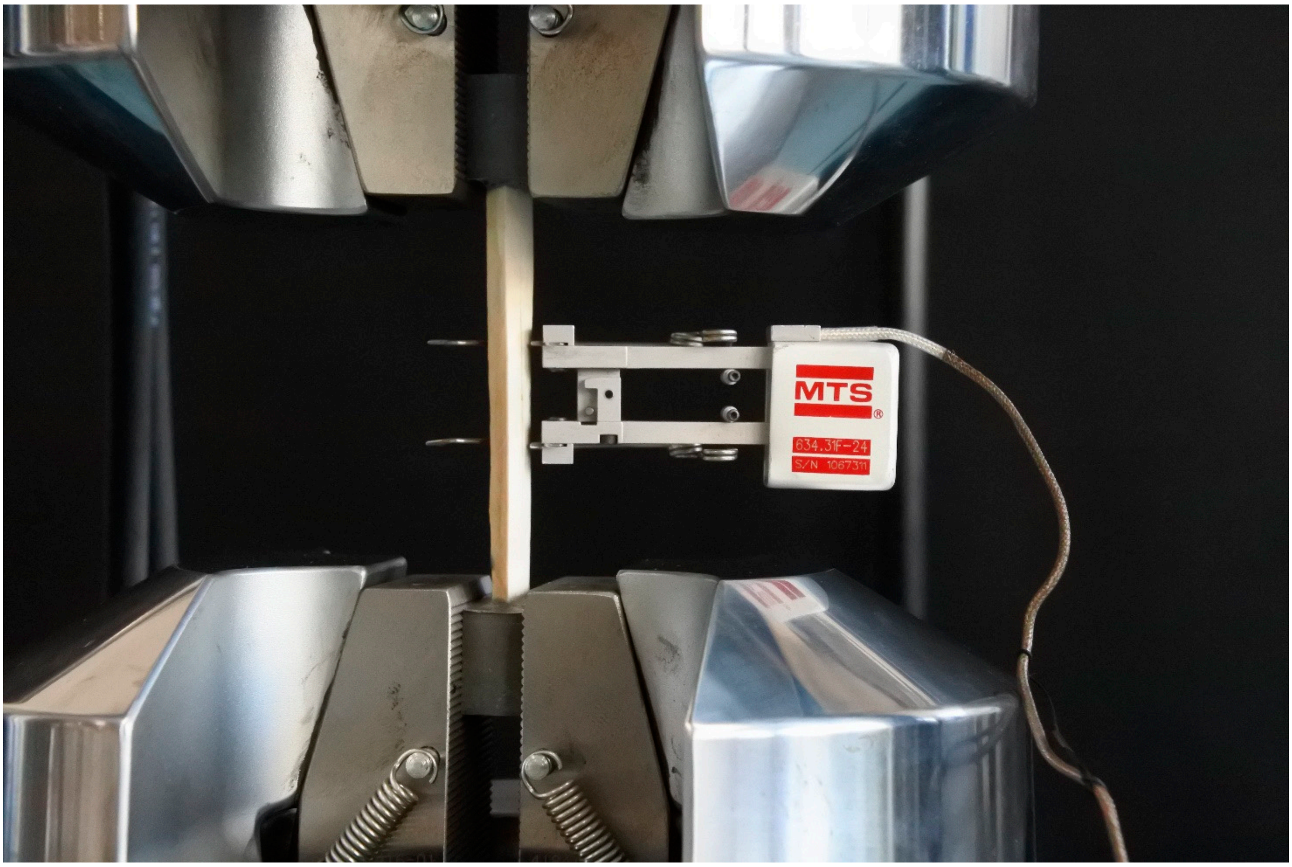

Figure S4: Sample 4.01. Image 1/4 of the uniaxial tensile test conducted on a sample of ostrich tibial shaft

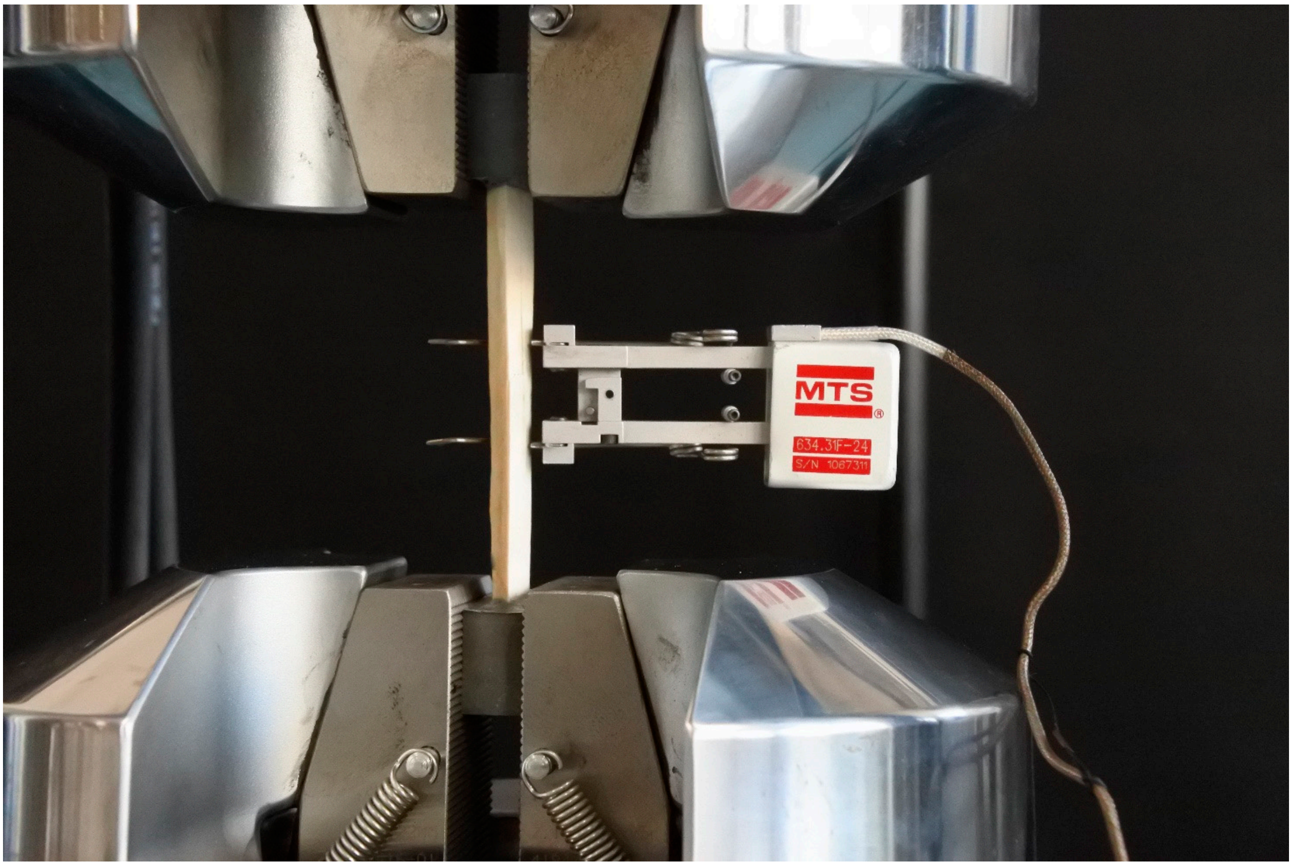

Figure S5: Sample 4.02. Image 2/4 of the uniaxial tensile test conducted on a sample of ostrich tibial shaft

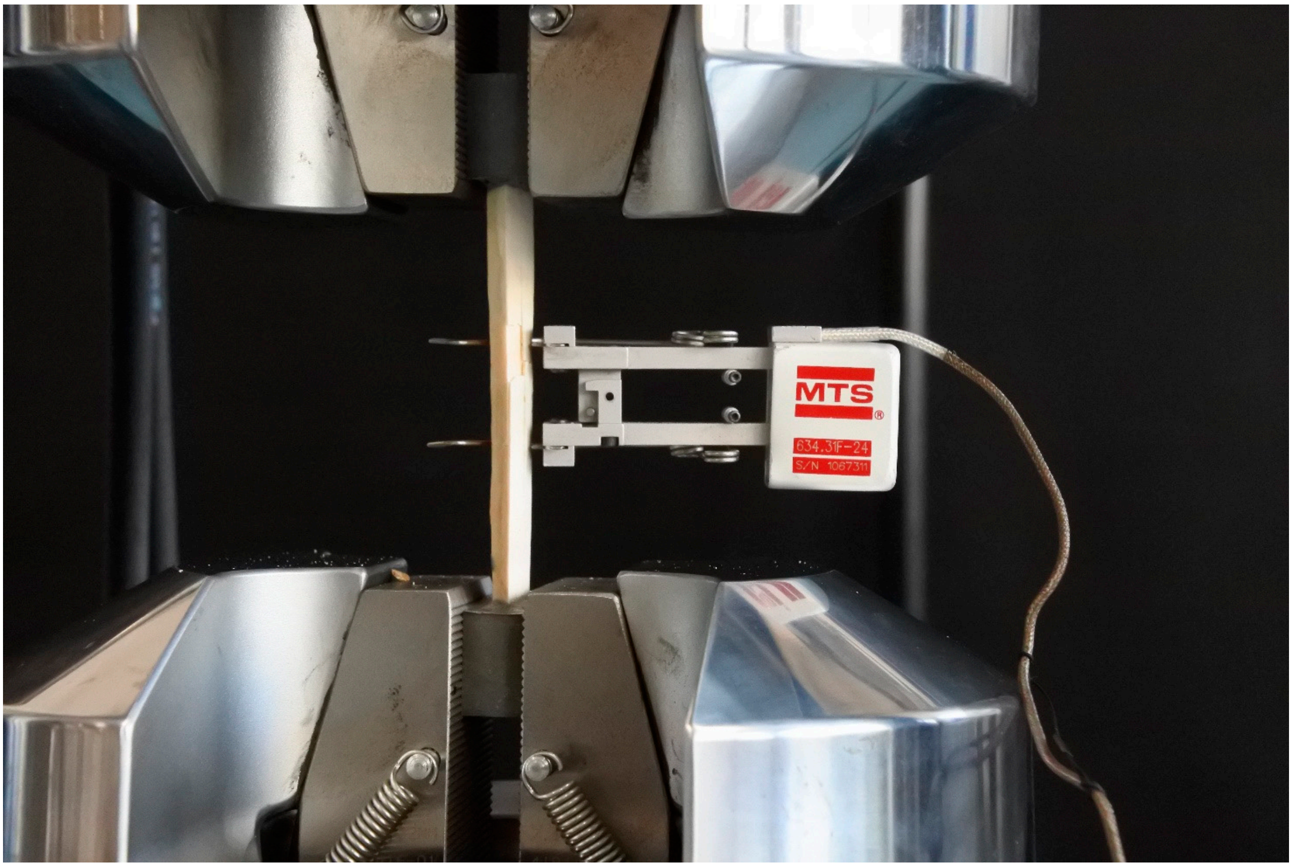

Figure S6: Sample 4.03. Image 3/4 of the uniaxial tensile test conducted on a sample of ostrich tibial shaft

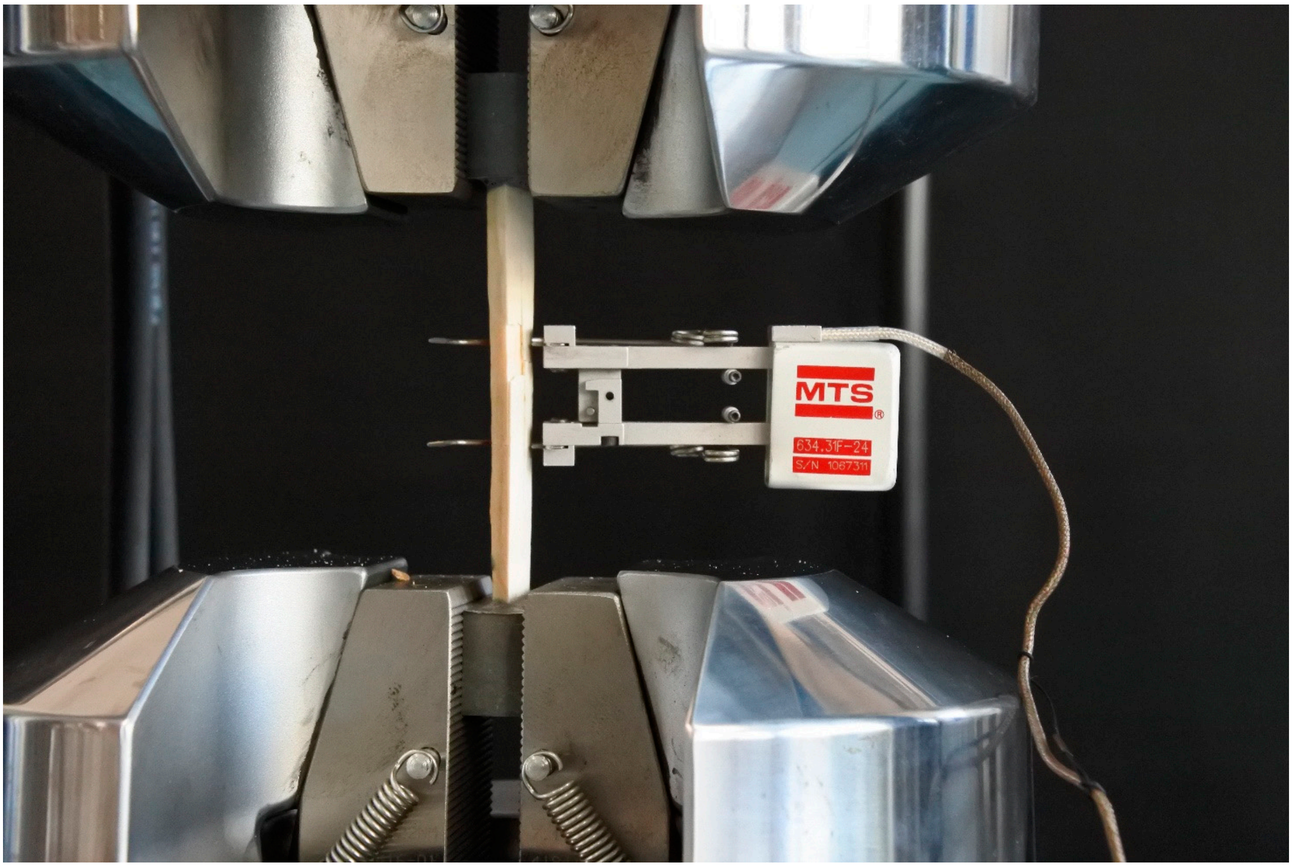

Figure S7: Sample 4.04. Image 4/4 of the uniaxial tensile test conducted on a sample of ostrich tibial shaft

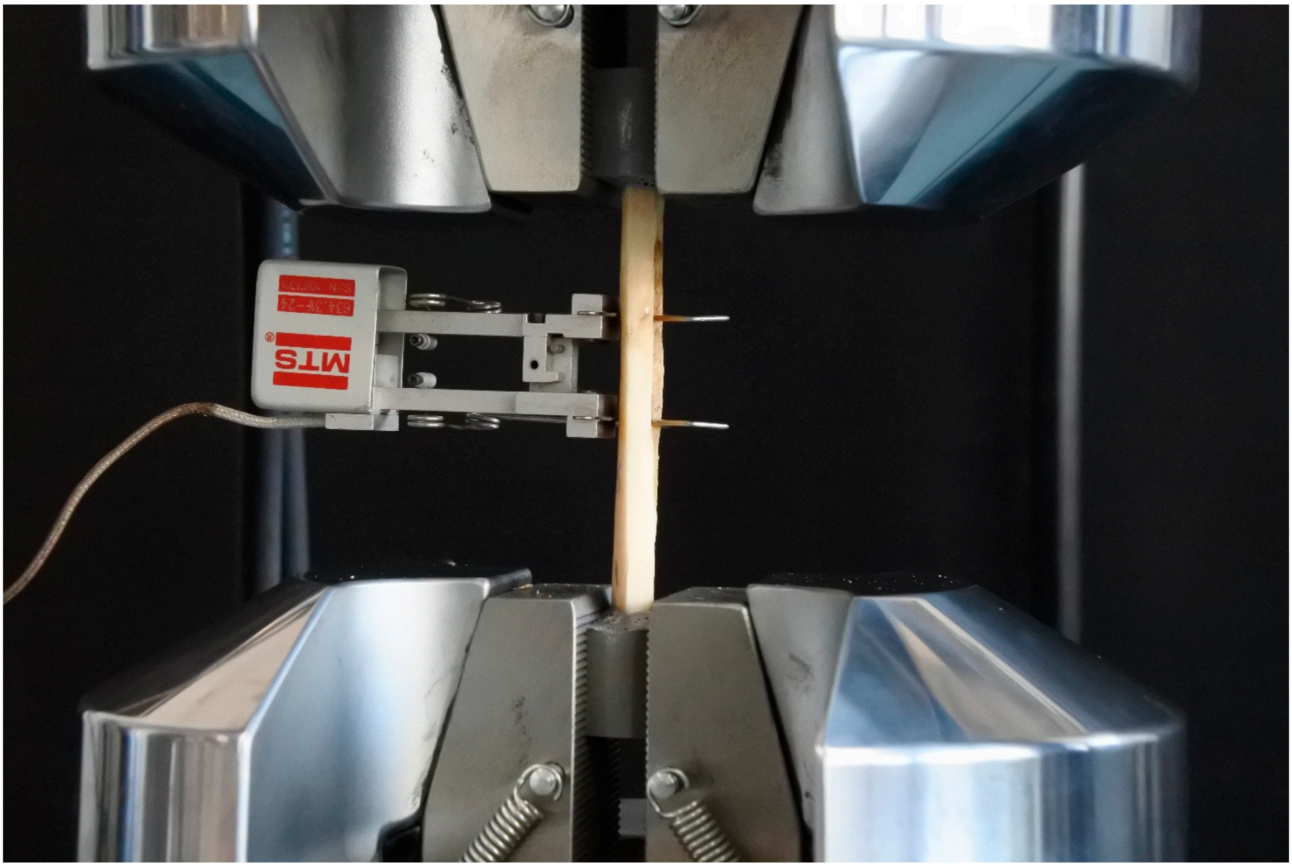

Figure S8: Sample 6.01. Image 1/7 of the uniaxial tensile test conducted on a sample of ostrich tibial shaft

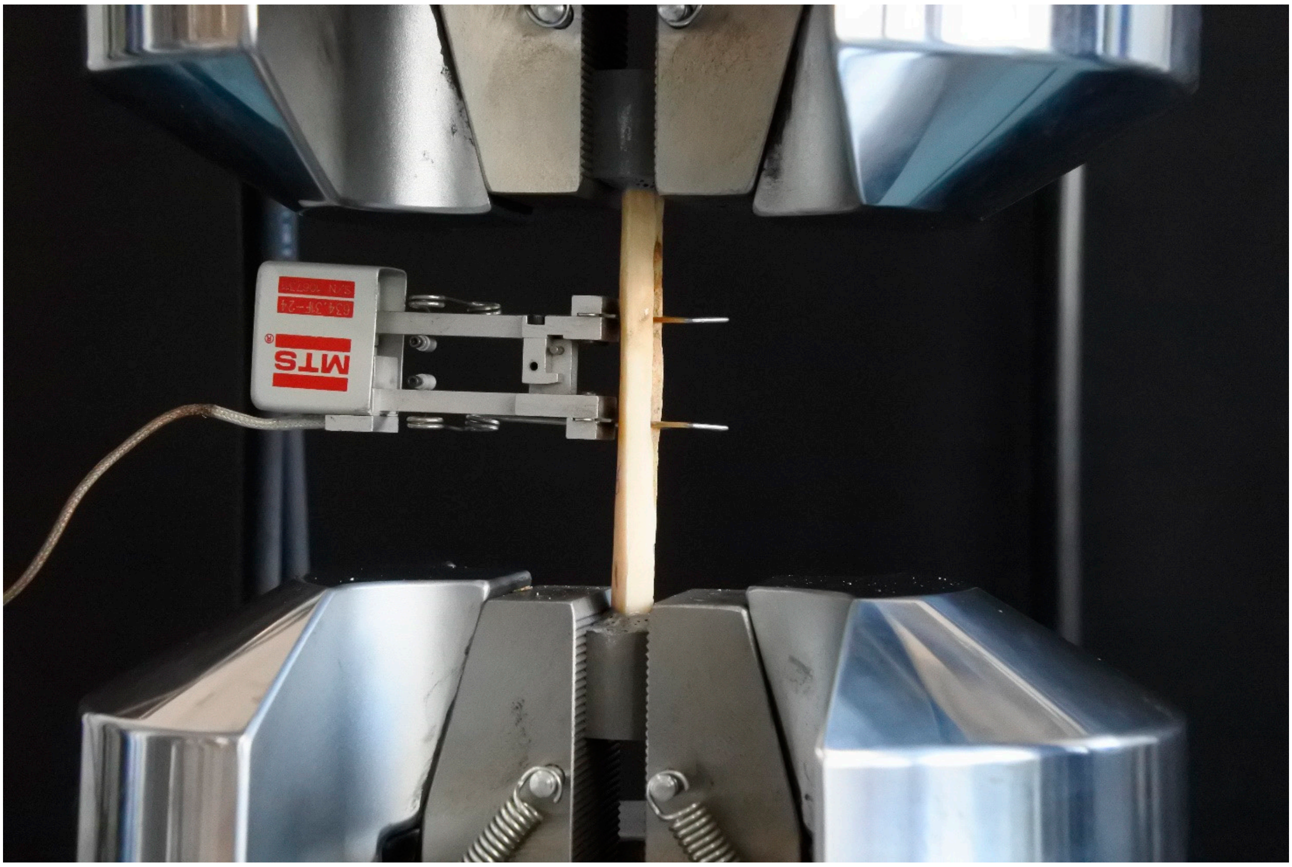

Figure S9: Sample 6.02. Image 2/7 of the uniaxial tensile test conducted on a sample of ostrich tibial shaft

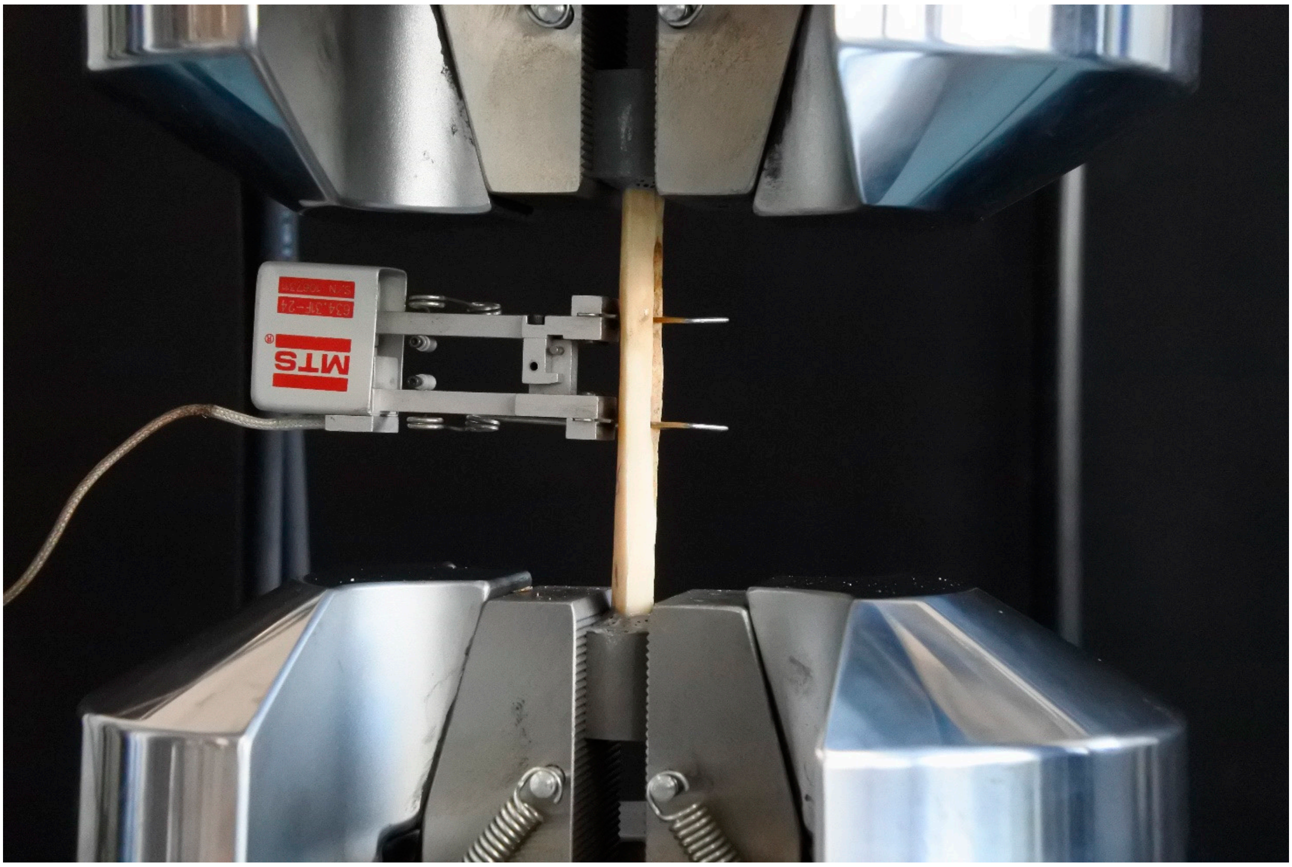

Figure S10: Sample 6.03. Image 3/7 of the uniaxial tensile test conducted on a sample of ostrich tibial shaft

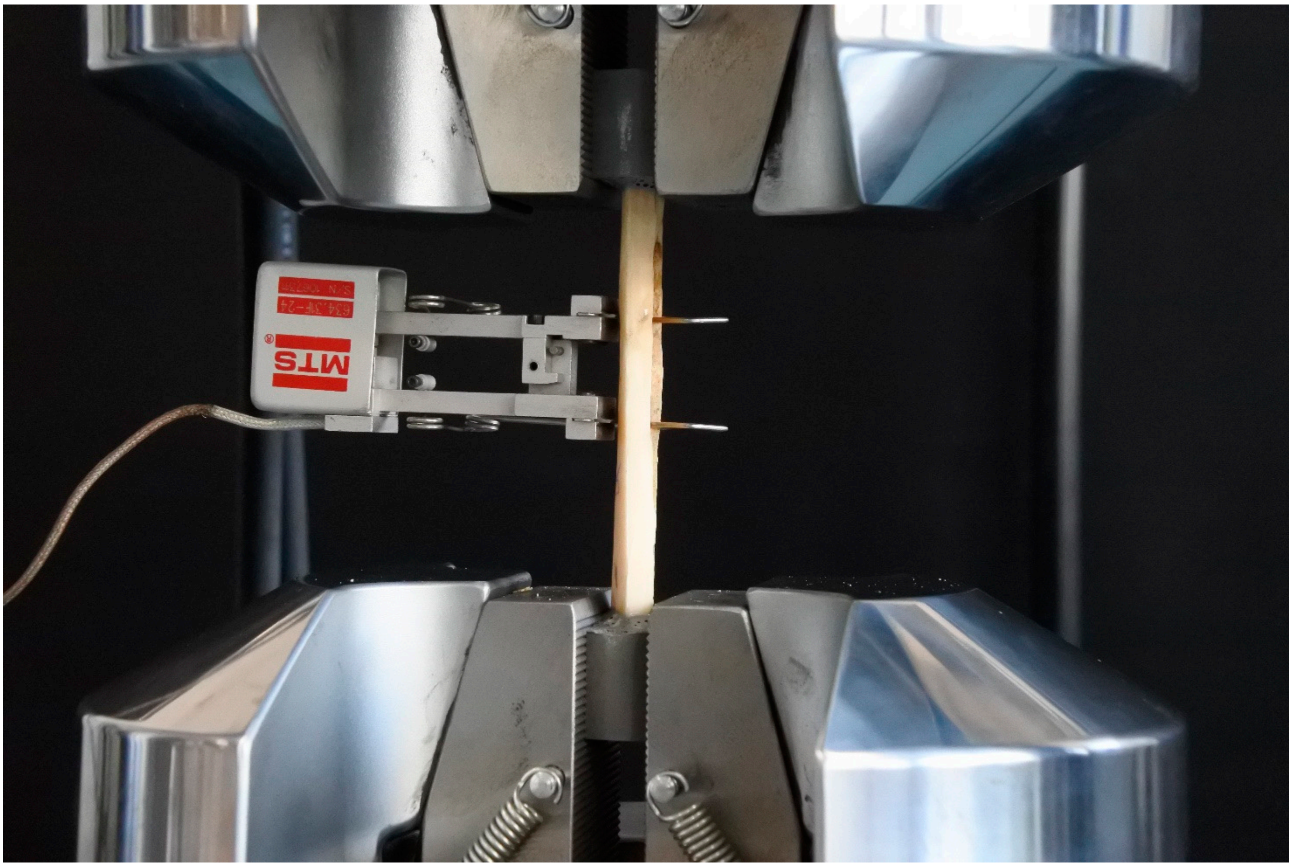

Figure S11: Sample 6.04. Image 4/7 of the uniaxial tensile test conducted on a sample of ostrich tibial shaft

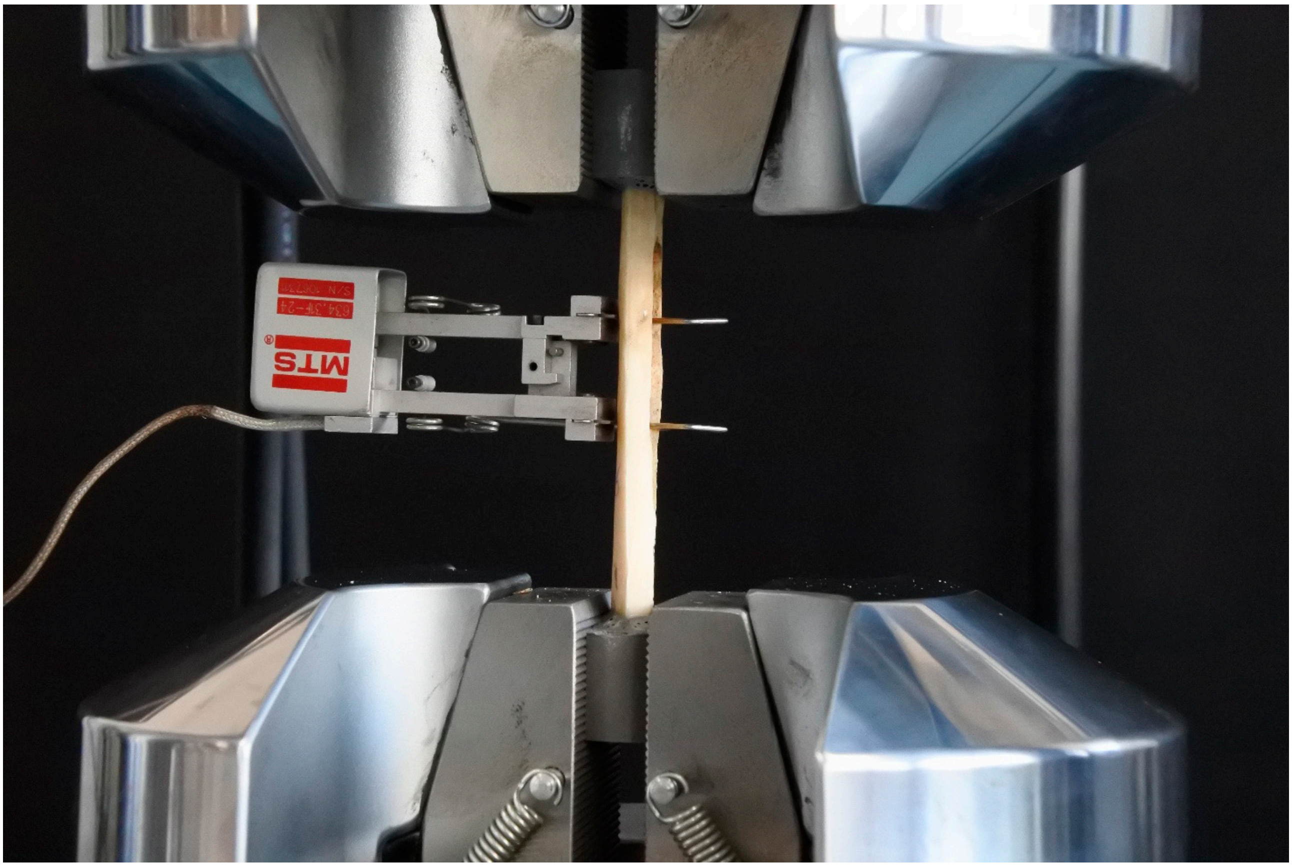

Figure S12: Sample 6.05. Image 5/7 of the uniaxial tensile test conducted on a sample of ostrich tibial shaft

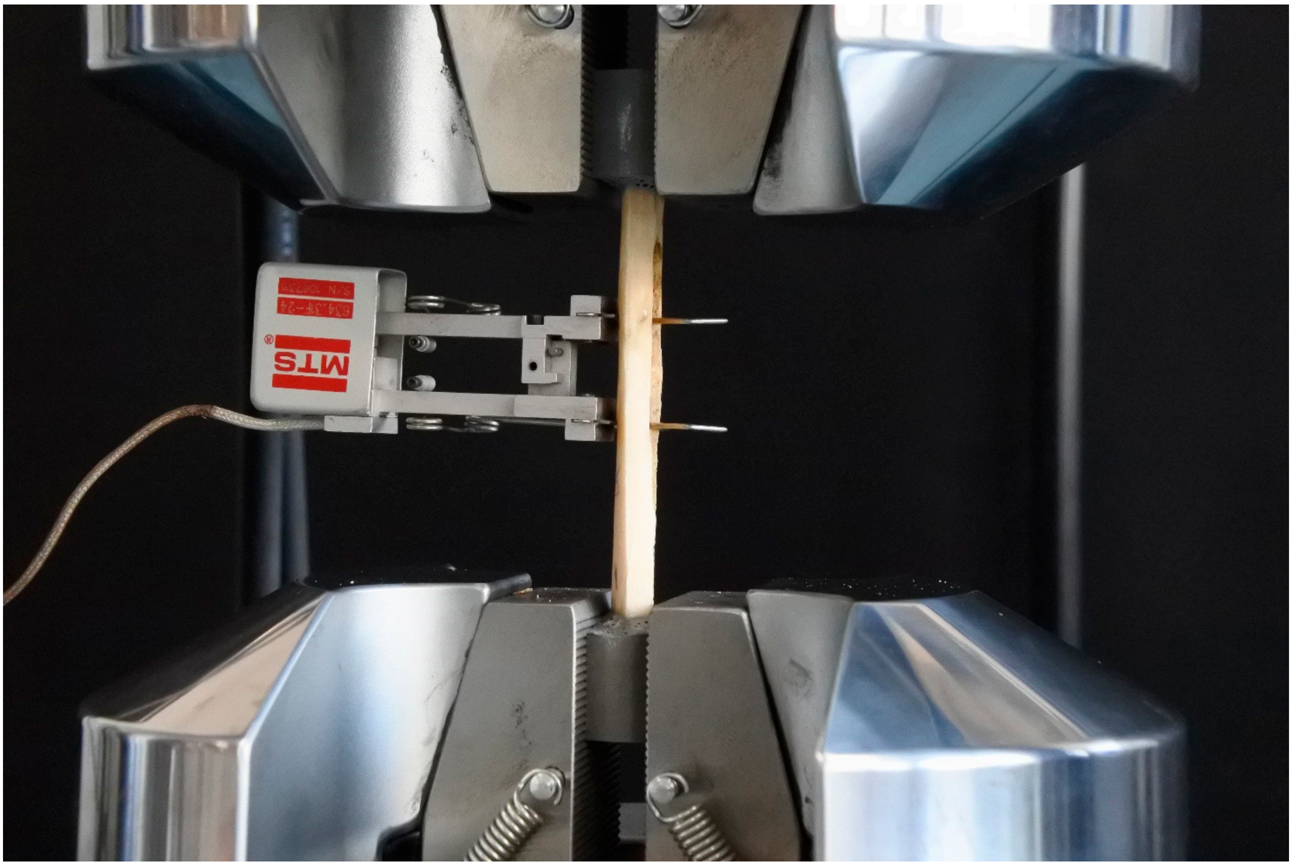

Figure S13: Sample 6.06. Image 6/7 of the uniaxial tensile test conducted on a sample of ostrich tibial shaft

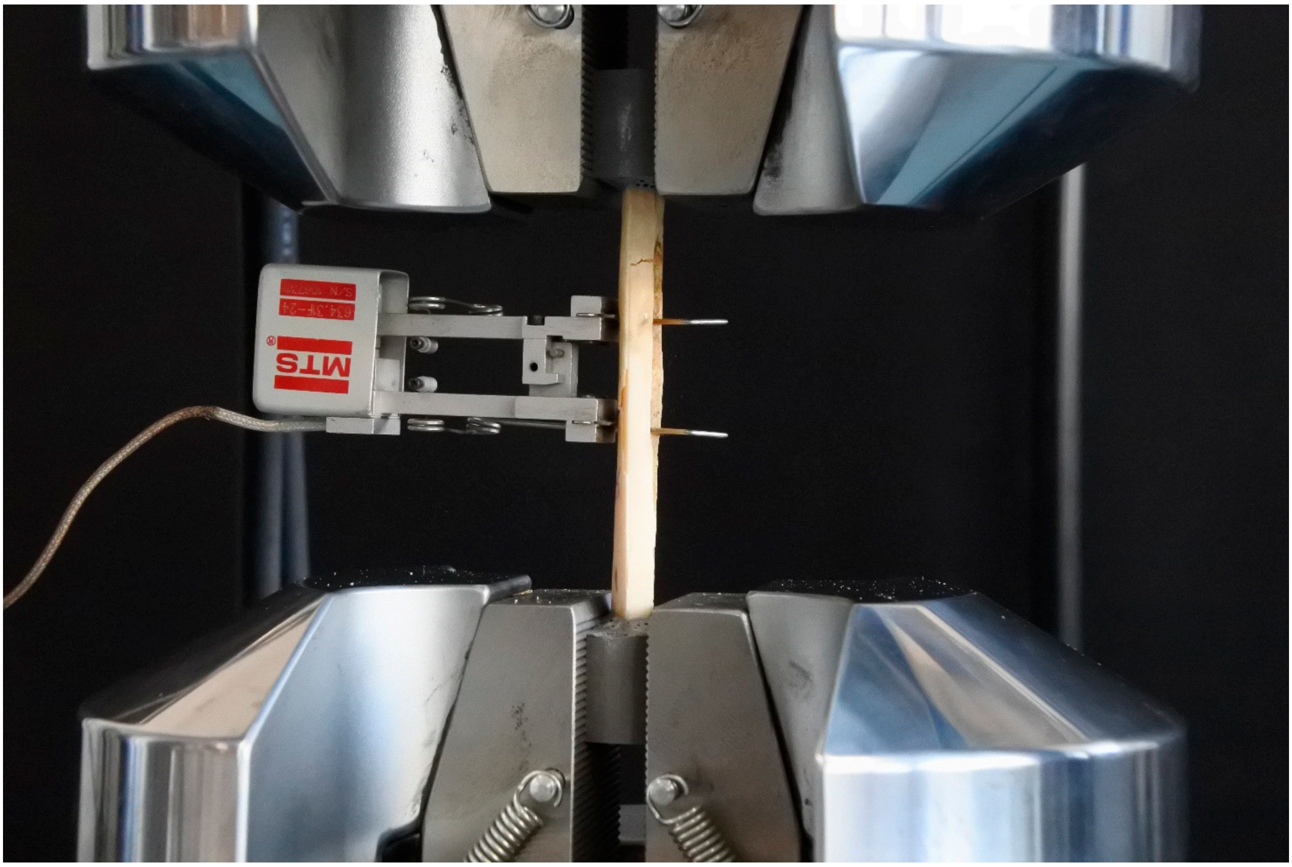

Figure S14: Sample 6.07. Image 7/7 of the uniaxial tensile test conducted on a sample of ostrich tibial shaft
